# Case report: Intra-abdominal inflammatory myofibroblastic tumor with mucinous features: a case of rapid recurrence and dissemination post-surgery

**DOI:** 10.3389/fonc.2024.1517710

**Published:** 2025-01-13

**Authors:** Xingchen Li, Jie Li, Chunxiao Liang, Qing Zou

**Affiliations:** Department of Radiology, People’s Hospital of Deyang City, Deyang, Sichuan, China

**Keywords:** inflammatory myofibroblastic tumor, recurrence, intra-abdominal, mucinous, computed tomography

## Abstract

Inflammatory myofibroblastic tumors (IMTs) are rare mesenchymal neoplasms with intermediate biological potential and are characterized by spindle-shaped myofibroblastic cells and significant inflammatory infiltrates. This case report describes a 24-year-old male with diabetes who was admitted to the hospital for over three days of vomiting and abdominal pain and was initially diagnosed with diabetic ketoacidosis. Upon admission, an abdominal CT scan revealed a large cystic-solid mass in the abdominal cavity and multiple nodules in the mesentery, omentum, and peritoneum, suggesting a preliminary diagnosis of an intra-abdominal mesenchymal tumor with peritoneal metastasis. The patient underwent tumor resection, and postoperative pathology confirmed it to be an IMT rich in mucin, with a Ki-67 proliferation index of 50%. Despite the initial symptom improvement after surgery, the patient experienced rapid recurrence with more extensive abdominal lesions. The patient refused further treatment, and died shortly thereafter. The case underscores the aggressive nature of inflammatory myofibroblastic tumors (IMTs) characterized by significant mucinous features, which are prone to recurrence and may suggest a poor prognosis. Radiological examinations and preoperative fine-needle aspiration biopsy may play a crucial role in managing such cases. Furthermore, alternative non-surgical treatment options or adjunct postoperative treatments could have a positive impact on the prognosis of this patient group. Further research is vital for enhancing our understanding of this rare tumor type and optimizing treatment strategies.

## Introduction

Inflammatory myofibroblastic tumors (IMTs) are rare mesenchymal neoplasms characterized by spindle-shaped, differentiated myofibroblastic cells and significant inflammatory infiltrates, predominantly plasma cells and/or lymphocytes. The WHO categorizes IMTs as having intermediate biological potential and is prone to local recurrence, yet with a low risk of distant metastasis (1). IMTs are commonly found in the lungs or abdominal soft tissues, particularly in children and young adults, with a median age of 9 years at diagnosis, but they can affect a wide range of anatomical sites and age groups (2, 3). The absence of distinctive features in laboratory and imaging studies makes the preoperative diagnosis of IMT challenging (4, 5). Consequently, the diagnosis relies heavily on histopathological and immunohistochemical examinations. Additional studies are needed to elucidate the full spectrum of IMT characteristics. This article describes a rare case of multiple mucinous IMT lesions in the mesentery, omentum, and peritoneum of the abdominal and pelvic cavity. To the best of our knowledge, only a few similar cases have been reported.

## Case presentation

A 24-year-old male presented to Deyang People’s Hospital with a three-day history of vomiting and abdominal pain. Over two years prior, he was admitted to our hospital’s basic surgery department for “epigastric pain and hematemesis” and was diagnosed with severe acute necrotizing pancreatitis and diabetes, with elevated blood glucose levels. During his hospital stay, he received insulin treatment, which was later switched to oral hypoglycemic drugs post-discharge. He reported fasting blood glucose levels of approximately 5-6 mmol/L and postprandial blood glucose levels below 10 mmol/L. Throughout his illness, the patient experienced significant weight loss, occasional limb numbness, and frothy urine, but no dry mouth, bitter taste, blurred vision, or alternating diarrhea and constipation. The patient discontinued hypoglycemic drugs for six months without subsequent blood glucose monitoring. Three days prior, he had experienced nausea, vomiting, and abdominal pain without an identifiable cause. His vomiting consisted of gastric contents, and the abdominal pain was intermittent, with some relief in the recumbent position. He exhibited no dizziness, cough, expectoration, chest tightness, or palpitations and was admitted to our emergency department. Emergency measurements revealed a blood glucose level of 27 mmol/L, blood ketones at 4 mmol/L, and POCT blood gas analysis with the following results: pH 7.02, HCO_3_- 1.90 mmol/L, BE(B) -27.00 mmol/L. The patient was admitted to the endocrinology department with a diagnosis of diabetic ketoacidosis.

**Figure 1:**
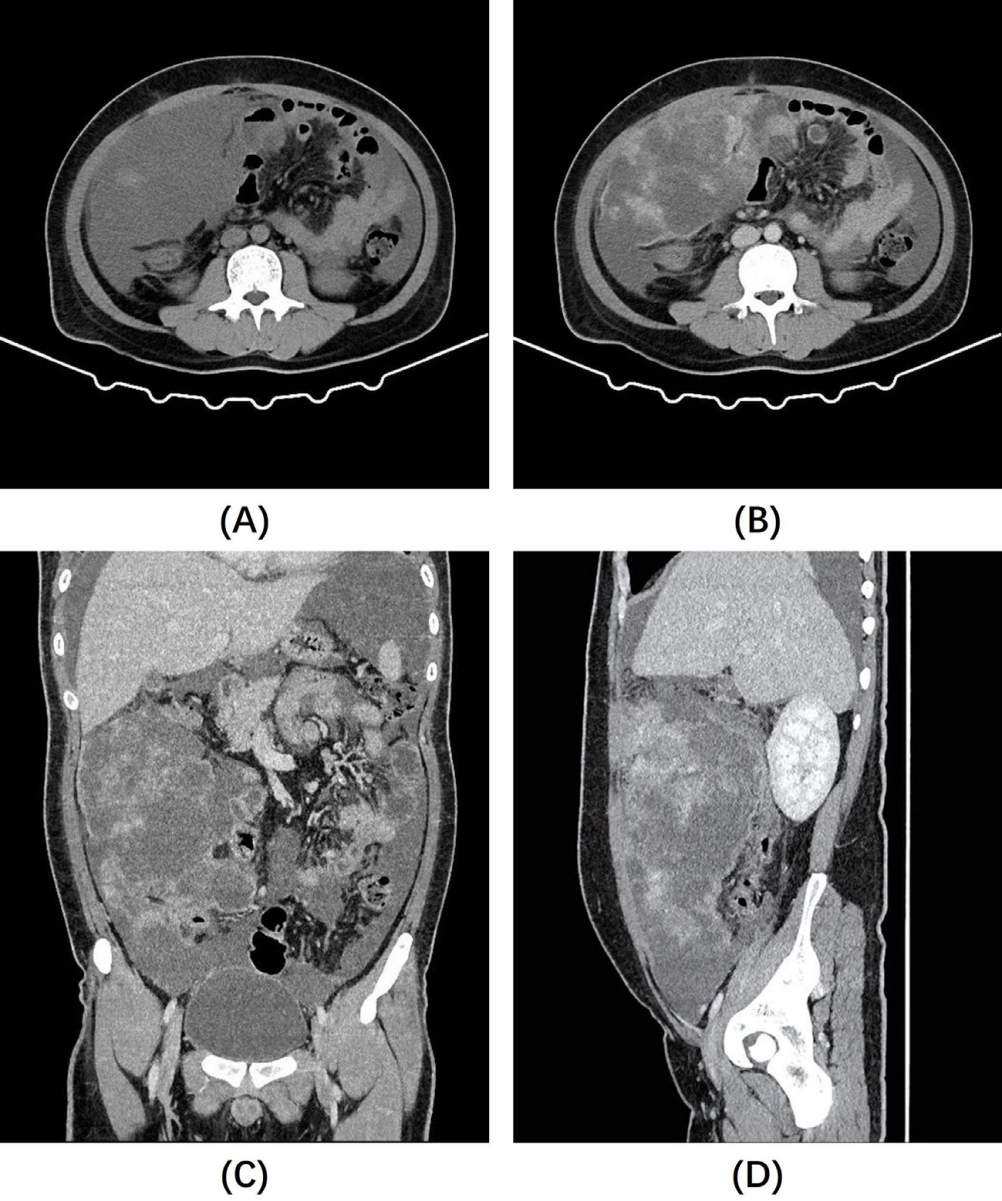
**(A)** Non-contrast abdominal CT images revealed a large, poorly defined mass of slightly lower density in the right abdominal cavity. **(B–D)** Post-contrast images showed heterogeneous enhancement of the mass, with significant non-enhancing areas and prominent enhancing foci that appear to “float” within them.

After admission, the patient underwent a contrast-enhanced CT scan of the entire abdomen, which revealed a large cystic-solid mass in the right mesenteric region and multiple nodules of varying sizes in the abdominal pelvic peritoneum and omentum. The solid components of the lesion exhibited progressive and significant contrast enhancement, with notable non-enhancing low-density areas. Within the larger right mesenteric lesion, enhancing lesions appeared to “float” within the low-density area (**Figure 1**). Given the close relationship of the lesions with the mesentery and peritoneum, and the absence of primary tumor signs elsewhere in the body, the condition was initially diagnosed as an intra-abdominal mesenchymal tumor with extensive peritoneal metastasis and implantation. However, making an accurate diagnosis based solely on imaging findings is challenging. The patient was transferred to the gastrointestinal surgery department of our hospital and underwent tumor resection. During surgery, a large tumor was found in the right abdominal cavity, about 20.0x15.0x8.0 cm, with an irregular shape, a bumpy surface, and a firm texture. Notably, the mass contained a significant amount of mucus, as confirmed by the postoperative pathology. Additionally, numerous tumor nodules (0.2 to 1.5 cm in diameter) were identified on various peritoneal surfaces, including the mesentery of the small intestine, posterior peritoneum, and mesocolon. The tumor in the right abdominal cavity was completely excised, and every effort was made to remove additional tumor nodules. Given the potential for residual tumor foci, the gastrointestinal surgeon suggested intraoperative placement of an intra-abdominal perfusion catheter for postoperative hyperthermic intraperitoneal perfusion therapy, which was ultimately declined by the patient’s family member.

**Figure 2:**
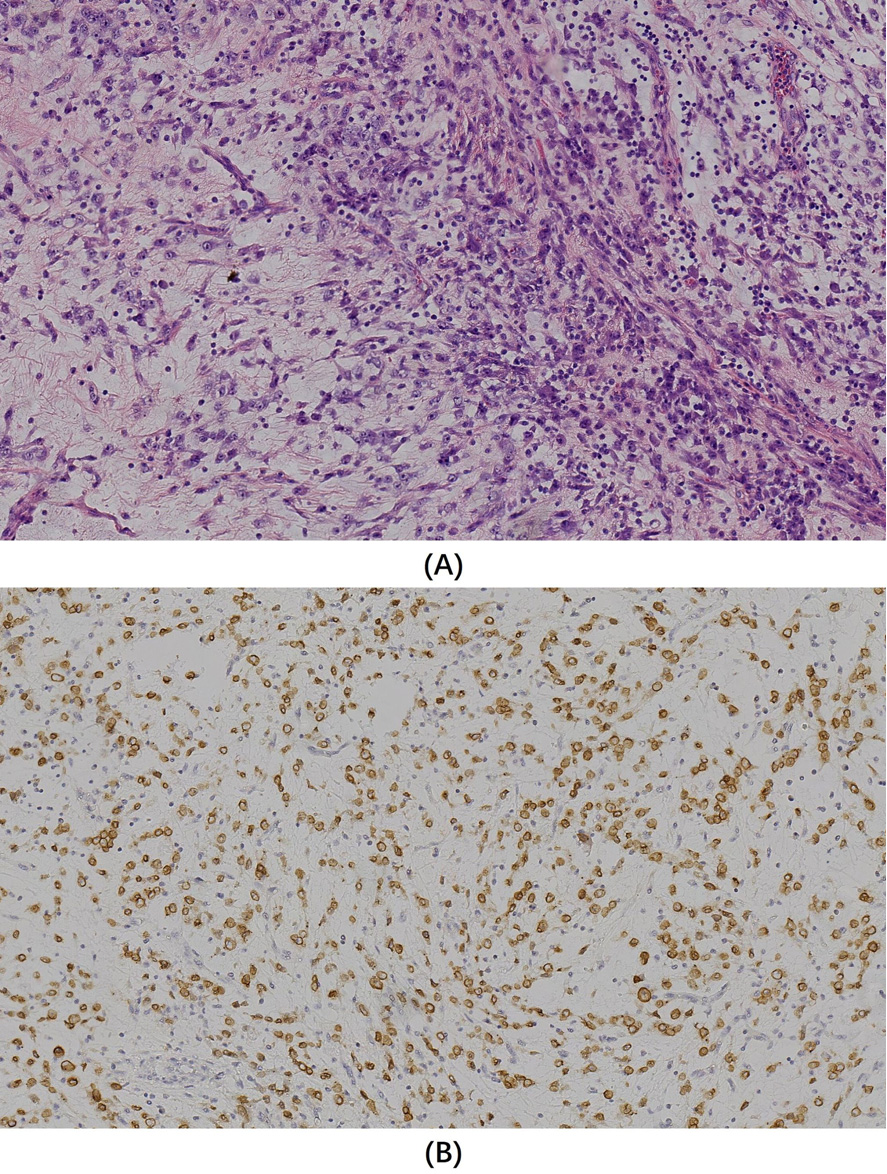
**(A)** Hematoxylin and eosin staining (original magnification x200) showed oval and spindle-shaped tumor cells with varying cellular densities against a prominent mucinous matrix background, accompanied by small blood vessels and marked inflammatory cell infiltration. **(B)** Immunohistochemistry for ALK (original magnification x200) revealed a diffuse membranous staining pattern in tumor cells.

Microscopy revealed oval- and spindle-shaped tumor cells with varying cellular densities against a prominent mucinous matrix background, accompanied by small blood vessels and marked inflammatory cell infiltration (**Figure 2A**). Postoperative immunohistochemical staining indicated diffuse positivity for Vimentin, Desmin, D2-40, and focal positivity for CK, SMA, and WT1 (Wilm’s tumor) in the tumor cells. The Ki-67 proliferation index was about 50%. Staining for S-100, NSE, Syn, EMA, SOX-10, Caldesmon, CD34, CD99, P63, Dog-1, CD117, HBME-1, and CK5/6 was negative. Immunohistochemistry for ALK demonstrated diffuse positive expression of ALK in the tumor cells, with a distinctive nuclear membranous staining pattern (**Figure 2B**).

**Figure 3:**
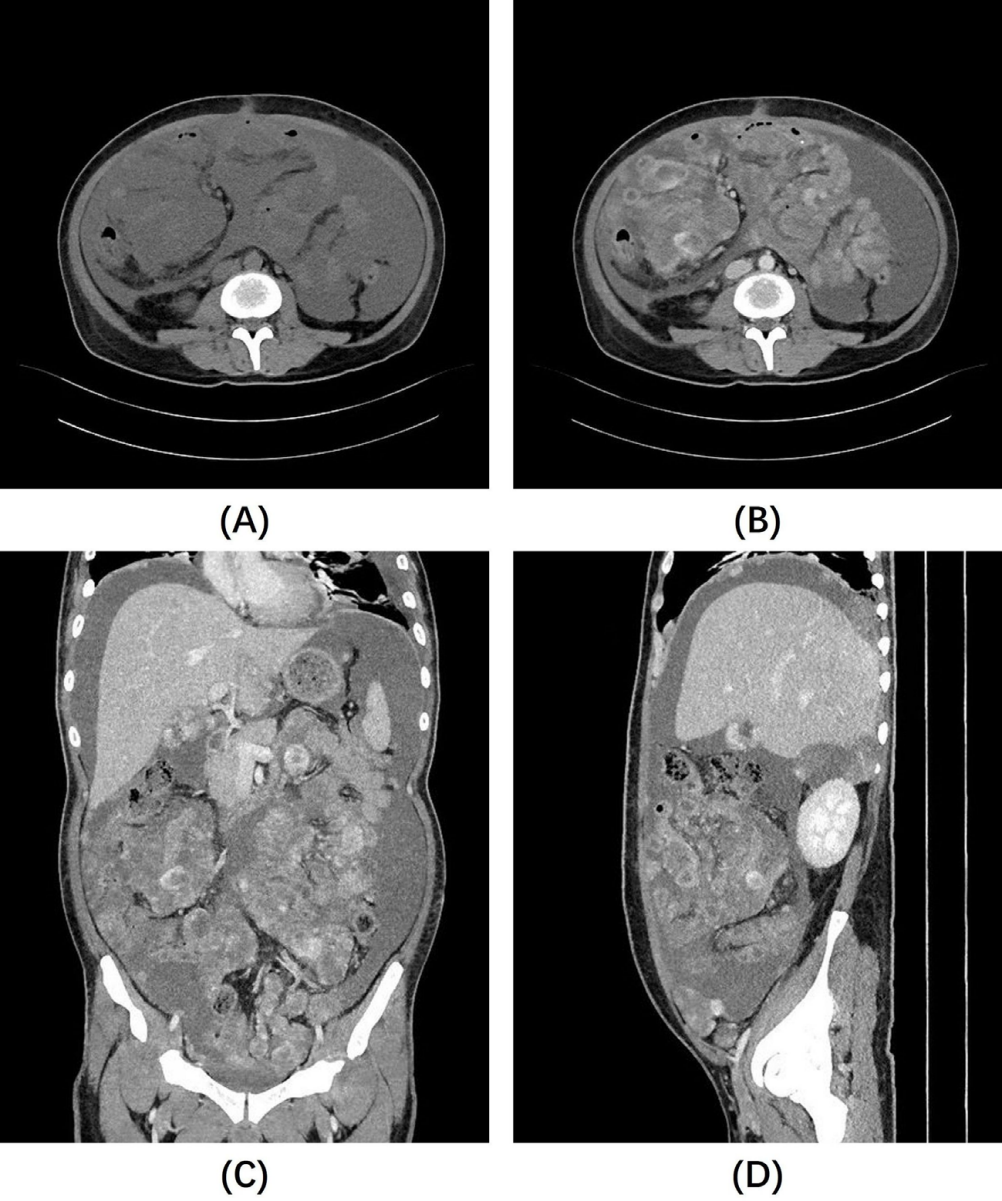
Follow-up abdominal CT scan: **(A)** Non-contrast abdominal CT images show a large amount of ascites, with unclear lesion contours. **(B–D)** Contrast-enhanced images reveal diffuse nodules and masses of varying sizes throughout the abdominal cavity, exhibiting markedly heterogeneous enhancement, with still noticeable non-enhancing areas, most of which are located at the center of the lesions.

The final pathological diagnosis, based on HE morphology and immunophenotypic results, confirmed the lesion as IMT. The patient’s symptoms improved postoperatively, leading to discharge. However, more than a month later, the patient returned to our hospital for a follow-up visit because of abdominal pain and bloating. A follow-up whole abdominal CT scan (**Figure 3**) showed multiple nodules and mass shadows of varying sizes in the abdominal mesenteric area, peritoneum, and omentum, which were significantly increased compared to the previous CT images, suggesting tumor recurrence and seeding metastasis. These lesions exhibit heterogeneous enhancement or more typical ring enhancement, but it is noteworthy that most of the lesions still show relatively obvious non-enhanced areas, suggesting the presence of mucus. The patient refused further treatment, and died a few days later.

## Discussion

IMT is a rare tumor with intermediate biological behavior, exhibiting local infiltration or recurrence, and rarely metastasizing. It can occur in nearly all organs and systems, including the lungs, mesentery, omentum, soft tissue, liver, spleen, pancreas, colon, spermatic cord, prostate, orbit, and peripheral and central nervous system, with the lungs, mesentery, and omentum being the most common sites (2). Clinical manifestations vary according to the anatomical location of the lesion. Abdominal IMT often presents with abdominal pain, fever, and weight loss, and sometimes with inflammatory signs such as fever, elevated erythrocyte sedimentation rate, and a marked increase in white blood cell count (> 80×10^9/L), all of which are nonspecific (6–8). Laboratory tests and imaging for IMT are nonspecific, complicating the preoperative diagnosis. The diagnosis of IMT primarily depends on histopathology and immunohistochemical analysis.

Some researchers have characterized IMT as an inflammatory process distinguished by the presence of myofibroblasts, histiocytes, plasma cells, and lymphocytes. These features indicate that IMT may represent a fibroinflammatory lesion that is associated with an extensive healing response to unidentified stimuli. Potential triggers include trauma, infection, inflammation, and surgery (9, 10). However, chromosomal rearrangements at 2p23 and ALK gene alterations are found in approximately 50% of IMT cases, implying a role for ALK in IMT pathogenesis and confirming its neoplastic nature (11).

Abdominal IMT exhibits diverse imaging features that are influenced by the affected organ. Hepatobiliary IMT often appears as single or multiple focal masses or as soft tissue infiltration around the portal vein, related or unrelated to focal lesions (12). IMT in hollow organs, such as the gastrointestinal tract, can show polypoid features, making it difficult to distinguish them from other tumors (13). Peritoneal, small intestine mesentery, or omental IMT can manifest as single or multiple lesions, isodense or hypodense to muscle, with larger lesions potentially necrotic centrally (7, 8, 14). The enhancement pattern correlates with histology; substantial fibrosis can lead to vascular scarcity and reduced delayed enhancement, whereas prominent inflammatory components show early enhancement, typically with central fibrosis and peripheral inflammation (15). Thus, IMT lesions typically exhibit early peripheral enhancements. The enhancement pattern also varies by IMT subtype; the myxoid-vascular type enhances more markedly, while the spindle cell-dense and fibrous types show less enhancement (13, 16).

Our case is unique because of the prominent cystic areas within the tumor, which become more evident as the lesion enlarges. These areas were slightly denser than water on CT scans and were not enhanced on contrast-enhanced images. Postoperative pathology confirmed the rich mucinous content of the lesion, suggesting that hypoenhanced areas within the IMT are not solely indicative of necrosis or fibrosis but may also represent mucinous components. Additionally, our case showed pronounced enhancement at the edges, similar to previously reported cases (17, 18). It is noteworthy that within the largest lesion, we observed enhanced foci “floating” in the low-density area, potentially linked to a rich mucinous edema background, which has rarely been mentioned in previous reports. We refer to this as the “enhanced floating sign”.

Unfortunately, our case recurred in the short term after surgery and presented with a more extensive lesion, most of which showed more typical ring enhancement and a central non-enhancing area. The tendency of recurrence and metastatic spread in our case may be attributed to several factors. First, the elevated ki67 index, which is approximately 50%, suggests a high rate of tumor cell proliferation. Second, the abundance of mucinous content and loose architecture of the tumor may also play a role. Other mucinous tumors in the abdomen, such as those of the appendix and ovaries, can also widely seed the peritoneum and produce a large amount of mucus; this is known as Pseudomyxoma Peritonei (PMP). PMP is distinguished by the redistribution of mucin and trapped neoplastic cells throughout the peritoneal cavity, leading to tumor accumulation at reabsorption sites, such as the omentum, paracolic gutters, and diaphragm underside (19). We speculate that a mechanism of tumor dissemination similar to PMP also exists in our case, given that our case also showed extensive peritoneal distribution and significant mucinous components, along with the appearance of more widespread lesions postoperatively. To our knowledge, there are currently scant studies exploring the correlation between significant mucinous features and the biological behavior of IMT. We posit that this may represent a novel avenue for future IMT research. Further validation of the impact of mucinous features on IMT’s biological behavior and imaging characteristics is necessary, particularly through larger cohort studies, to yield new insights for diagnostic and therapeutic strategies.

Due to the uncertainty of diagnosis, the risk of disease progression, and the lack of reliable alternative treatment options, complete surgical resection is generally considered the preferred and recommended treatment method for IMT (2, 20, 21). Surgical treatment is effective for focal lesions; however, in cases like ours, simple tumor resection may not improve survival rates and could potentially worsen the condition. In addition to surgery, radiotherapy, chemotherapy, targeted therapy, immunotherapy, and anti-inflammatory treatments have also been proposed for the treatment of IMT (7, 22). An existing study has indicated that for the more aggressive pathological subtype of IMTs, epithelioid inflammatory myofibroblastic sarcoma (EIMS), treatment with ALK inhibitors can yield a prognosis similar to that of non-EIMS (23). Meanwhile, fine-needle aspiration biopsy is considered a reasonable diagnostic method in appropriate clinical settings (24). Preoperative fine-needle aspiration biopsy to determine the pathological histological subtype and degree of cell proliferation activity of such tumors, combined with imaging studies to interpret the distribution and imaging characteristics of the lesions, can better inform clinical treatment decisions. Because for individuals battling recurrent or inoperable IMTs, it is essential to explore adjunct postoperative treatments or nonsurgical options.

## Conclusion

In this study, we described a rare case of multifocal intra-abdominal malignant IMT, characterized by a significant mucinous component with conspicuous non-enhancing hypodense areas and “floating” enhanced foci within them on contrast-enhanced CT images. We have introduced the term “enhanced floating sign” to describe this imaging feature, underscoring the presence of mucin-rich tumor lesions. Unfortunately, the patient experienced rapid recurrence of the tumor and extensive intra-abdominal seeding metastasis after surgery. While surgical resection remains the most effective treatment for IMT, alternative non-surgical treatments or adjunct postoperative treatments could potentially improve the prognosis for patients with intra-abdominal IMTs like ours. Radiological examinations and preoperative fine-needle aspiration biopsies are expected to play a crucial role in managing such cases. However, larger-scale studies are required to confirm these findings.

## Data Availability

The original contributions presented in the study are included in the article/supplementary material. Further inquiries can be directed to the corresponding author.

## References

[1] Coffin CM Fletcher JA (2019). “Inflammatory Myofibroblastic Tumor,” in World Health Organization Classification of Tumours: Pathology and Genetics of Tumours of Soft Tissue and Bone, eds. Fletcher CDM Unni KK Mertens F (Lyon: IARC Press) (2002), 91–93.

[2] Fu GX Xu CC Yao NF Gu JZ Jiang HL Han XF . Inflammatory myofibroblastic tumor: A demographic, clinical and therapeutic study of 92 cases. Math Biosci Eng. (2019) 16:6794–804. doi: 10.3934/mbe.2019339 31698588

[3] Raitio A Losty PD . Treatment and outcomes in pediatric inflammatory myofibroblastic tumors - A systematic review of published studies. Eur J Surg Oncol. (2024) 50:108388–8. doi: 10.1016/j.ejso.2024.108388 38713995

[4] Liu F Hu H-J Wang J-K Li F-Y . Inflammatory myofibroblastic tumor of the liver: challenges in the preoperative diagnosis and treatment. J Gastrointest Surg. (2017) 22:1132–3. doi: 10.1007/s11605-017-3637-1 29235003

[5] G HA Kumar S Singla S Kurian N . Aggressive inflammatory myofibroblastic tumor of distal pancreas: A diagnostic and surgical challenge. Cureus. (2022) 14(3):e22820. doi: 10.7759/cureus.22820 35399449 PMC8980218

[6] Tan H Wang B Xiao H Lian Y Gao J . Radiologic and clinicopathologic findings of inflammatory myofibroblastic tumor. J Comput Assist Tomogr. (2016) 41:90–7. doi: 10.1097/rct.0000000000000444 27224222

[7] Shatzel J Wooten K Ankola A Cheney RT Morrison CD Skitzki JJ . Inflammatory myofibroblastic tumor of the mesentery: a clinical dilemma. Int J Clin Oncol. (2011) 17(4):380–4. doi: 10.1007/s10147-011-0297-0 21823041

[8] Lu C-H Huang H-Y Chen H-K Chuang J-H Ng S-H Ko S-F . Huge pelvi-abdominal Malignant inflammatory myofibroblastic tumor with rapid recurrence in a 14-year-old boy. World Journal of Gastroenterology (World Journal of Gastroenterology). (2010) 16(21):2698–701. doi: 10.3748/wjg.v16.i21.2698 PMC2880786 20518095

[9] Gupta S Goyal P Yang Y Fitzgerald W . Tracheal inflammatory myofibroblastoma: A rare tumor of the trachea. Cureus. (2019) 11(4):e4484. doi: 10.7759/cureus.4484 31259102 PMC6581385

[10] Kubal C Ghotkar S Gosney J Carr M . Pleural inflammatory myofibroblastoma: a locally aggressive intra-thoracic tumour. Journal of Cardiothoracic Surgery. (2007) 2(1):29. doi: 10.1186/1749-8090-2-29 17598912 PMC1914059

[11] Su L Atayde-Perez A Sheldon S Fletcher J Sw W . Inflammatory myofibroblastic tumor: cytogenetic evidence supporting clonal origin. Modern Pathol. (1998) 11(4):364–8. 9578087

[12] Venkataraman S Semelka RC Braga L Danet I-M Woosley JT . Inflammatory myofibroblastic tumor of the hepatobiliary system: report of MR imaging appearance in four patients. Radiology. (2003) 227:758–63. doi: 10.1148/radiol.2273020572 12728186

[13] Horger M Pfannenberg C Bitzer M Wehrmann M Claussen CD . Synchronous gastrointestinal and musculoskeletal manifestations of different subtypes of inflammatory myofibroblastic tumor: CT, MRI and pathological features. European Radiology. (2004) 15(8):1713–6. doi: 10.1007/s00330-004-2453-7 15322807

[14] Choi AH Bohn OL Beddow TD McHenry CR . Inflammatory myofibroblastic tumor of the small bowel mesentery: an unusual cause of abdominal pain and uveitis. JOURNAL OF GASTROINTESTINAL SURGERY. (2011) 15(4):584–8. doi: 10.1007/s11605-010-1408-3 21207180

[15] Kirchgesner T Danse E Sempoux C Annet L Dragean CA Trefois P . Mesenteric inflammatory myofibroblastic tumor: MRI and CT imaging correlated to anatomical pathology. JBR-BTR. (2015) 97(5):301–2. doi: 10.5334/jbr-btr.1335 25597213

[16] Zhao XT Yue SW Cheng Q Liu P Chang LY Zhao XX . CT findings of inflammatory myofibroblastic tumor of different pathological types. Zhonghua Yi Xue Za Zhi. (2017) 97:43–6. doi: 10.3760/cma.j.issn.0376-2491.2017.01.011 28056290

[17] Furukawa Y Kitajima K Komoto H Zenitani M Oue T Yokoyama H . CT and MRI findings of inflammatory myofibroblastic tumor in the bladder. Case Reports in Oncology. (2022) 15(1):120–5. doi: 10.1159/000521921 PMC8921957 35350811

[18] Saleem MI Ben-Hamida MA Barrett AM Bunn SK Huntley L Wood KM . Lower abdominal inflammatory myofibroblastic tumor -an unusual presentation- a case report and brief literature review. European Journal of Pediatrics. (2006) 166(7):679–83. doi: 10.1007/s00431-006-0305-y 17109166

[19] Carr NJ . New insights in the pathology of peritoneal surface Malignancy. J Gastrointest Oncol. (2021) 12:S216–29. doi: 10.21037/jgo-2020-01 PMC8100698 33968439

[20] Gleason BC Hornick JL . Inflammatory myofibroblastic tumours: where are we now? J Clin Pathol. (2007) 61:428–37. doi: 10.1136/jcp.2007.049387 17938159

[21] Fragoso AC Eloy C Estevão-Costa J Campos M Farinha N Lopes JM . Abdominal inflammatory myofibroblastic tumor a clinicopathologic study with reappraisal of biologic behavior. J Pediatr Surg. (2011) 46:2076–82. doi: 10.1016/j.jpedsurg.2011.07.009 22075336

[22] Chmiel P Słowikowska A Banaszek Ł Szumera-Ciećkiewicz A Szostakowski B Spałek MJ . Inflammatory myofibroblastic tumor from molecular diagnostics to current treatment. Oncol Res. (2024) 32:1141–62. doi: 10.32604/or.2024.050350 PMC11209743 38948020

[23] Liu X Gong C Zhang J Feng W Guo Y Sang Y . Clinicopathological analysis and treatment of adult patients with inflammatory myofibroblastic tumor: A 15-year single-center study. Cancer Res Treat. (2023) 55:1001–10. doi: 10.4143/crt.2022.894 PMC10372594 36915248

[24] Stoll LM Li QK . Cytology of fine-needle aspiration of inflammatory myofibroblastic tumor. Diagn Cytopathol. (2010) 39:663–72. doi: 10.1002/dc.21444 20730898

